# Migrasome and Tetraspanins in Vascular Homeostasis: Concept, Present, and Future

**DOI:** 10.3389/fcell.2020.00438

**Published:** 2020-06-16

**Authors:** Yaxing Zhang, Jing Wang, Yungang Ding, Jiongshan Zhang, Yan Xu, Jingting Xu, Shuhui Zheng, Hongzhi Yang

**Affiliations:** ^1^Department of Traditional Chinese Medicine, The Third Affiliated Hospital, Sun Yat-sen University, Guangzhou, China; ^2^Department of Ophthalmology, Qingdao Fubai Eye Hospital, Qingdao, China; ^3^Department of Ophthalmology, Qingdao Ludong Eye Hospital, Qingdao, China; ^4^Department of Gastrointestinal Endoscopy, Guangzhou Cadre Health Management Center/Guangzhou Eleventh People’s Hospital, Guangzhou, China; ^5^Biofeedback Laboratory, Xinhua College of Sun Yat-sen University, Guangzhou, China; ^6^Research Center for Translational Medicine, The First Affiliated Hospital, Sun Yat-sen University, Guangzhou, China

**Keywords:** cell migration, migrasome, migracytosis, tetraspanins, vascular homeostasis

## Abstract

Cell migration plays a critical role in vascular homeostasis. Under noxious stimuli, endothelial cells (ECs) migration always contributes to vascular repair, while enhanced migration of vascular smooth muscle cells (VSMCs) will lead to pathological vascular remodeling. Moreover, vascular activities are involved in communication between ECs and VSMCs, between ECs and immune cells, et al. Recently, Ma et al. (2015) discovered a novel migration-dependent organelle “migrasome,” which mediated release of cytoplasmic contents, and this process was defined as “migracytosis.” The formation of migrasome is precisely regulated by tetraspanins (TSPANs), cholesterol and integrins. Migrasomes can be taken up by neighboring cells, and migrasomes are distributed in many kinds of cells and tissues, such as in blood vessel, human serum, and in ischemic brain of human and mouse. In addition, the migrasome elements TSPANs are wildly expressed in cardiovascular system. Therefore, TSPANs, migrasomes and migracytosis might play essential roles in regulating vascular homeostasis. In this review, we will discuss the discoveries of migration-dependent migrasome and migracytosis, migrasome formation, the basic differences between migrasomes and exosomes, the distributions and functions of migrasome, the functions of migrasome elements TSPANs in vascular biology, and discuss the possible roles of migrasomes and migracytosis in vascular homeostasis.

## Blood Vessels and Vascular Homeostasis

The vasculature is one of the first functional organs to form during embryogenesis and matures into a closed cardiovascular system, adding up to about 90,000 km in total length in adults (Eelen et al., 2018). Structurally, blood vessels are primarily made up of three layers: *tunica interna* (*intima*), *tunica media* (*media*), and *tunica externa* (*adventitia*), which is a network of connective tissue, including collagen fibers, fibroblasts, *vasa vasorum*, nerve endings, progenitor/stem cells, myofibroblasts, pericytes, lymphocytes, macrophages, and dendritic cells et al. (Moos et al., 2005; Hu and Xu, 2011; Campbell et al., 2012; Wilting and Chao, 2015; Halper, 2018; Zhang Y. et al., 2018); while lymphatic capillaries are thin-walled vessels of approximately 30–80 μm in diameter, composed of a single layer of oak-leaf-shaped lymphatic ECs that differ in many ways from blood vascular ECs (Alitalo, 2011). Almost all tissues, except for cartilage, cornea and lens et al., in the body rely on blood vessels for a continuous supply of nutrients and oxygen, and on lymphatic vessels to collect excess protein-rich fluid that has extravasated from blood vessels and transport it back into blood circulation, and these vessels provide gateways for immune surveillance (Alitalo, 2011; Potente et al., 2011; Eelen et al., 2018). Additionally, blood vessels take part in controlling systemic pH and temperature homeostasis (Eelen et al., 2018).

During adult life, the maintenance of vascular homeostasis is the result of balancing vascular damage and injury with repair and regeneration, while also integrating environmental cues to optimize vascular function and blood vessel growth, thus ensuring adequate supply of oxygen and nutrients to tissues, and maintaining other functions mentioned above (Marsboom and Rehman, 2018). Vascular remodeling denotes morphological changes and reorganization of vessel wall structure, morphologically, all three layers of the arterial wall are concurrently affected by neointimal hyperplasia, medial thickening, and adventitial fibrosis attributable to the interaction of leukocyte recruitment, VSMCs accumulation, and endothelial recovery, in response to various noxious stimuli, such as hemodynamic stress, mechanical injury, inflammation, or hypoxia et al. (Schober, 2008; Zhang et al., 2016, 2017). All these lead to decrease in cross-sectional vessel diameters and increase in the thickness of the arterial wall. Therefore, vascular homeostasis maintenance is an active process, involved in the growth, migration and death of vascular cells, activation of immune cells in vasculature, as well as the generation and degradation of ECM, all these coordinate with environmental cues to maintain the function of blood vessels (Moos et al., 2005; Liu et al., 2014; Zhang and Dong, 2014; Zhang and Li, 2017).

## Cell Migration in Development, Immune Defense and Vascular Homeostasis

Cell migration plays an essential role in a variety of physiological and pathological processes (Le Clainche and Carlier, 2008). During developmental processes, cell migration is fundamental to the establishment of the embryonic architecture like gastrulation (Keller, 2005), and migration is also required for neural crest cells colonization (Szabo and Mayor, 2018). Recently, *Yu Li* group indicated that migration-dependent migrasomes release developmental cues, including Cxcl12, into defined locations in embryos to modulate organ morphogenesis during zebrafish gastrulation (Jiang et al., 2019). The immune defensive function of most immune cells depends on their ability to migrate through complex microenvironments, either randomly to patrol for the presence of antigens or directionally to reach their next site of action (Moreau et al., 2018). In cardiovascular system, ECs migration occurs during vasculogenesis and angiogenesis, and also in damaged vessels to restore vessel integrity, VSMCs migrate to the intima and proliferate to contribute to neointimal lesions under pathophysiological conditions (Michaelis, 2014; Wang et al., 2015; Zhang et al., 2016). Therefore, cell migration is the key event during the regulation of vascular homeostasis.

## The Discoveries of Migration-Dependent Migrasome and Migracytosis

Porter et al. (1945) and Taylor and Robbins (1963) have observed the long projections from the surface of cells, and long tubular structures as migrating cells retracted from the substratum, respectively. Oppenheimer and Humphreys (1971), Culp and Black (1972), and Terry and Culp (1974) isolated the specific macromolecules, which remain on substrates after treating with chelating agents. Morphological and biochemical analyses showed that these SAMs are finger-like extensions and contain relatively large amounts of cell surface components that participate in cell adhesion, such as fibronectin, proteoglycans, and gangliosides (Rosen and Culp, 1977; Culp et al., 1979; Rollins and Culp, 1979; Murray and Culp, 1981; Mugnai et al., 1984; Barletta et al., 1989). SAMs are analogous to the retraction fibers of migrating cells that are also enriched with TSPANs (Penas et al., 2000; Zhang and Huang, 2012; Yamada et al., 2013). Besides TSPANs, SAMs also contain large amounts of TSPANs associated proteins, but not focal adhesion proteins, and thus resemble the footprints (Yamada et al., 2013). Although these structures wildly present in different cell types, however, they have received little attention, their structure, characterization and function are less well-known.

Ma et al. (2015) observed and characterized an extracellular membrane-bound vesicular structure, which are PLSs relate to cells. They found that a cell will leave retraction fibers behind it, and vesicles grow on the tips or at the intersections of retraction fibers during the process of migration; eventually, the retraction fibers break up, and PLSs, as a package of vesicles and cytosolic contents enclosed within a single limiting membrane, are released into the medium or directly taken up by surrounding cells (da Rocha-Azevedo and Schmid, 2015; Ma et al., 2015). The formation of these PLSs is dependent on both migration and actin polymerization, thus, they named these PLSs “migrasomes,” which average lifespan is about 400 min, this migration-dependent release mechanism is named “migracytosis” (Ma et al., 2015).

## The Molecular Mechanism of Migrasomes Formation

As migrasomes are membrane structures, therefore, membrane-localized proteins and the organization of membrane are essential for migrasomes formation. TSPANs family, which includes 33 members in human beings (Hemler, 2008; Rubinstein, 2011), are abundant in membranes of various types of endocytic organelles and in exosomes (Zoller, 2009; van Niel et al., 2018), and are also essential components of migrasomes (Table 1). TSPANs contain a number of shared structural features, including TM1, TM2, TM3 and TM4, a very short intracellular loop (typically four amino acids) between TM2 and TM3, a short ECL1 between TM1 and TM2, a large ECL2 between TM3 and TM4, short amino- and carboxy-terminal tails, and a large central pocket inside the intramembranous region bounded by the four transmembrane helices (Hemler, 2005; Zoller, 2009; Zimmerman et al., 2016; Umeda et al., 2020). The Glu219 in TSPAN28 is the critical residue for cholesterol molecule binding at the central cavity, TSPAN10 also possesses a polar residue in this position, while most other TSPANs have a polar residue one helical turn earlier, with other cholesterol-binding residues highly conserved throughout evolution, offering a potential mechanism for how TSPANs might detect cholesterol or other membrane lipids (Zimmerman et al., 2016). The TEMs, including TSPANs, a set of TSPANs-associated proteins and a high concentration of cholesterol, is a functional unit in cell plasma membranes (Yanez-Mo et al., 2009; Huang et al., 2019). The physiological and pathological functions of TSPANs family genes have been investigated and confirmed by the established TSPANs KO mice (Supplementary Table 1).

**TABLE 1:** The basic characteristics of migrasomes and exosomes.

Indexes	Exosomes	Migrasomes	References
Diameters	30–200 nm	0.5–3 μm	Ma et al., 2015; Pegtel and Gould, 2019
Contents	Membrane organizers, enzymes, lipids, chaperon proteins, intracellular trafficking proteins, cell adhesion proteins, signal transduction proteins, cell-type-specific proteins, biogenesis factors, histones, nucleic acids (DNA: mtDNA, dsDNA, ssDNA, viral DNA; RNA: mRNA, miRNA, Pre-miRNA, Y-RNA, circRNA, mtRNA, tRNA, tsRNA, snRNA, snoRNA, piRNA), amino acids, glycoconjugates, and metabolites et al.	Vesicles, membrane proteins, contractile proteins, cytoskeleton proteins, enzymes, chaperon proteins, vesicle traffic proteins, receptors, cell adhesion proteins, extracellular proteins, DNA or RNA binding proteins, complement system proteins, signal transduction proteins, lipids, et al.	Ma et al., 2015; Schmidt-Pogoda et al., 2018; van Niel et al., 2018; Pegtel and Gould, 2019; Kalluri and LeBleu, 2020
TSPANs profiles	TSPAN6, 8, 24, 25, 26, 27, 28, 29, 30, et al.	TSPAN4, TSPAN7, et al.	Ma et al., 2015; van Niel et al., 2018; Jiang et al., 2019; Pegtel and Gould, 2019
Classical membrane markers	TSPAN28, TSPAN29, TSPAN30, TSG101, et al.	TSPAN4, TSPAN7, Integrin α5 and β1, et al.	Ibrahim and Marban, 2016; Wu et al., 2017; Jiang et al., 2019
Specific protein markers	SUMF2, LAMP1	NDST1, PIGK, CPQ, EOGT	Ma et al., 2015; Ibrahim and Marban, 2016; Zhao et al., 2019
Release	By fusion of MVBs with plasma membrane	By breaking the retraction fibers	Ma et al., 2015

*mtDNA, mitochondrial DNA; dsDNA, double-stranded DNA; ssDNA, single-stranded DNA; miRNA, microRNA; circRNA, circular RNA; mtRNA, mitochondrial RNA; tRNA, transfer RNA; tsRNAs, tRNA-derived small RNAs; snRNA, small nuclear RNA; snoRNA, small nucleolar RNA; piRNA, PIWI-interacting RNA; TSG101, tumor susceptibility gene 101; SUMF2, sulfatase modifying factor 2; LAMP1, lysosomal associated membrane protein 1; NDST1, bifunctionalheparan sulfate N-deacetylase/N-sulfotransferase 1, PIGK, phosphatidylinositol glycan anchor biosynthesis, class K, CPQ, carboxypeptidase Q; EOGT, EGF domain-specific O-linked N-acetylglucosaminetransferase; MVB, multivesicular bodies.*

During the discovery of migrasomes, *Yu Li* group identified that TSPAN4 is abundant in migrasomes membrane, and acts as the clearest migrasomes marker (Ma et al., 2015). Overexpression of TSPAN1, 2, 3, 4, 5, 6, 7, 9, 13, 18, 25, 26, 27, and 28 enhance the formation of migrasomes, and TSPAN1, 2, 4, 6, 7, 9, 18, 27, and 28 have strong effects (Huang et al., 2019). KO of TSPAN4 impairs migrasomes formation in MGC-803 cells and NRK, while deficiency of TSPAN4 in L929 cells did not impair migrasomes formation, presumably due to the presence of other migrasomes-forming TSPANs (Huang et al., 2019). TSPAN4, TSPAN7, cholesterol and integrins are necessary for migrasomes formation (Wu et al., 2017; Huang et al., 2019; Jiang et al., 2019). TSPAN4 in migrasomes is about four times higher than in retraction fibers, cholesterol is enriched about 40-fold in migrasomes relative to retraction fibers, and integrins are highly enriched on migrasomes and are only present at very low levels on retraction fibers (Wu et al., 2017; Huang et al., 2019). The activated integrin α5 is mainly enriched on the bottom side of migrasomes while TSPAN4 is on the upper side (Wu et al., 2017). Mechanistically, when a cell migrates, integrins enable cell migration and the correct pairing of integrin with its specific ECM partner protein provides the adhesion for retraction fiber tethering, and retraction fibers are formed at the back of the migrating cells (Wu et al., 2017). The mechanical stress exerted along the retraction fibers triggers clustering of TSPANs, such as TSPAN4, TSPAN7, and cholesterol molecules, leading to the formation of “TEMAs,” which enriched causes stiffening of the plasma membrane, thus, facilitating a new migrasome formation (Huang et al., 2019; Jiang et al., 2019; Tavano and Heisenberg, 2019).

## The Basic Differences Between Migrasomes and Exosomes

Both migrasomes and exosomes are the extracellular membrane-bound vesicular structures, however, the comparison of migrasomes and exosomes proteomics indicates that the two structures share only 27% (158) proteins, and there is still much difference among size, contents, TSPANs expression profiles, classical membrane markers, specific protein markers, and the process of release (da Rocha-Azevedo and Schmid, 2015; Ma et al., 2015; Ibrahim and Marban, 2016; Wu et al., 2017; Chen et al., 2018; Schmidt-Pogoda et al., 2018; van Niel et al., 2018; Huang et al., 2019; Jeppesen et al., 2019; Jiang et al., 2019; Pegtel and Gould, 2019; Zhao et al., 2019; Kalluri and LeBleu, 2020; Table 1).

Based on these above, TSPAN4/7 and integrin α1, α3, α5, β1 et al., which are expressed in the membrane of migrasomes, can act as essential structure markers for migrasomes (Ma et al., 2015; Wu et al., 2017; Schmidt-Pogoda et al., 2018; Huang et al., 2019; Jiang et al., 2019). NDST1, PIGK, CPQ and EOGT, which are enriched in migrasomes, but are absent or barely detectable in exosomes, are specific protein markers for migrasomes (Zhao et al., 2019). The transmission electron microscopy is used to detected ultrastructure of migrasomes *in situ* within cultured cells (Chen et al., 2018). A recent study by *Yu Li* group indicated that WGA is a probe for convenient, rapid detection of migrasomes in both fixed and living cells (Chen et al., 2019). The detail methods for visualizing migrasomes have also been described in two articles of “Detection of Migrasomes” and “WGA is a probe for migrasomes” by *Yu Li* group (Chen et al., 2018, 2019).

## The Distributions and Functions of Migrasomes

**FIGURE 1:**
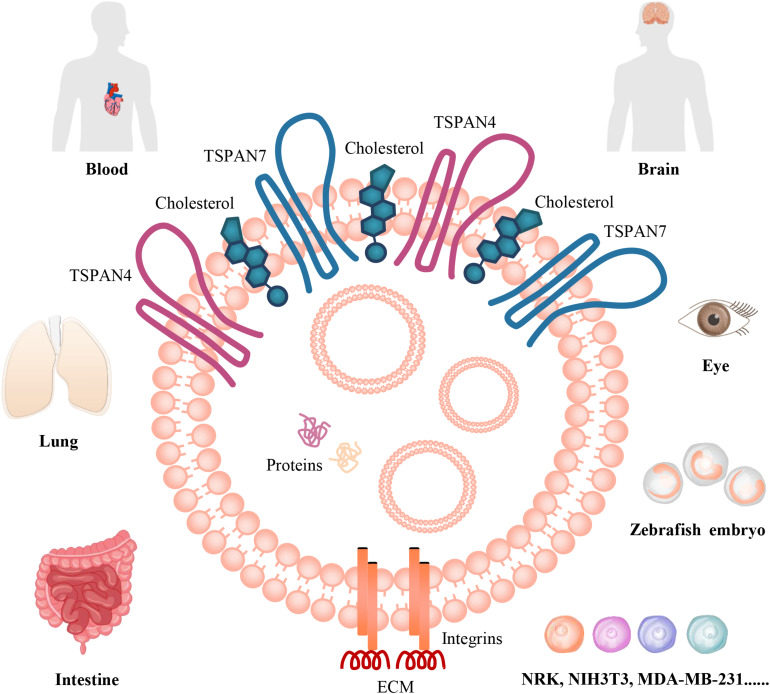
The organization and distributions of migrasomes. Migrasome, which is an extracellular membrane-bound vesicular structure and includes cytosolic proteins and small vesicles in the cavity, is organized by TSPANs, cholesterol, and integrins. The matching of specific integrin-ECM pairs determine when and where migrasome can be generated *in vivo*. They are distributed in human blood and stroke specimens; in mouse brain and eye, rat lung, rat/mouse intestine; in zebrafish embryo. They are also found in numerous cancer cells, such as MDA-MB-231, SKOV-3, HCT116, SW480, MGC803, B16; in normal rat or mouse cells, including NRK, NIH3T3, mouse embryonic stem cells, hippocampal neurons and bone marrow-derived macrophages, and microglia; and in HaCaT *in vitro*.

Studies in the past 5 years indicated that migrasomes are distributed in many cell types *in vitro*, including NRK, mouse embryonic fibroblasts (MEF and NIH3T3), mouse melanoma cell (B16), mouse embryonic stem cells, mouse hippocampal neurons and mouse bone marrow-derived macrophages, and murine microglia, human keratinocyte (HaCaT), human breast cancer cell (MDA-MB-231), human colon cancer cell (HCT116), human adenocarcinoma cell (SW480), human gastric carcinoma cell (MGC803), human ovarian adenocarcinoma cell (SKOV-3) (Ma et al., 2015; Schmidt-Pogoda et al., 2018). Moreover, migrasomes are also distributed in various organs, such as human stroke specimens, mouse eye, rat lung, rat/mouse intestine (Ma et al., 2015; Schmidt-Pogoda et al., 2018; Figure 1). They tend to be present inside cavity such as pulmonary alveoli or blood vessel, for example, in intestine, migrasomes are inside blood capillaries or lymph capillaries, in the lamina propria of ileum crypts, or in connective tissue (Ma et al., 2015). The mice model study indicate that migrasomes can be detected in the brain of the ischemic hemispheres of mice feeding standard diet, and high-salt diet induces massive migrasomes formation in microglia/macrophages and leads to reduce numbers of anti-inflammatory F4/80^+^-microglia/macrophages and astrocytes after cerebral ischemia (Schmidt-Pogoda et al., 2018). F4/80^+^-migrasomes are co-localized with NeuN, which is expressed in nuclei and cytoplasm of neurons, and there is a significant correlation between the extent of migrasome formation and the number of shrunk neurons (Schmidt-Pogoda et al., 2018). These findings suggest that migrasome, which incorporates and dispatches the cytosol of surrounding neurons, might act as a novel, sodium chloride-driven mechanism in acute ischemic stroke pathophysiology (Schmidt-Pogoda et al., 2018). However, the precise regulatory mechanisms between migrasome formation and neuronal loss still need further investigation. Recently, the physiological roles of migrasomes in living animals has been investigated by using the zebrafish embryo as a model system, the results indicated that migrasomes were enriched on a cavity underneath the embryonic shield where they served as chemoattractants to ensure the correct positioning of dorsal forerunner cells vegetally next to the embryonic shield, thus coordinating organ morphogenesis during zebrafish gastrulation (Jiang et al., 2019). Surprisingly, *Yu Li* group found that migrasomes are present in human serum, but their origin and function are not clear (Zhao et al., 2019). Therefore, to discuss and investigate its roles in vascular biology is an interesting topic.

## The Current Understanding of Tetraspanins Family in Vascular Homeostasis

Migracytosis, a cell migration-dependent mechanism for releasing intracellular contents into external environment, and migrasomes, the vesicular structures that mediate migracytosis, are involved in cell–cell communications, as that the releasing contents can be taken up by surrounding cells (Ma et al., 2015; Chen et al., 2018). Based on their distribution *in vivo* and *in vitro*, it seems that they may play essential roles in maintaining vascular homeostasis. However, there are few literatures discussed the functions of migrasomes and migracytosis in blood vessel. Therefore, to understand the vascular function of TSPANs, which are the key elements of migrasomes, may help us to investigate and understand the possible roles of migrasomes and migracytosis in vascular homeostasis in the near future.

Bailey et al. (2011) have investigated TSPANs expression at the mRNA level in human ECs by analyzing largescale transcriptomic data from publicly available SAGE experiments. They found that HUVECs expressed 23 TSPANs and human liver endothelium expressed 17 TSPANs. The RNA-seq analyses and RNA-arrays for TSPANs family genes expressed in HCASMCs, MASMCs and mouse carotid arteries indicate that all TSPANs except for TSPAN16 and TSPAN19 are detectable in these cells and tissue (Zhao et al., 2017). More than one half of TSPANs family genes are significantly reduced by TGF-β1, while only *TSPAN2*, *12*, *13*, and *22* are up-regulated, and *TSPAN2* shows the most dramatic change by TGF-β1 (Zhao et al., 2017). Myocardin, which is another master regulator of VSMCs differentiation, increases 8 *TSPANs* genes expression, including *TSPAN2, 7, 10, 11, 12, 15, 21, 33*, and *TSPAN2* showed the greatest up-regulation (Zhao et al., 2017). The expression profiles of TSPANs family genes in ECs and VSMCs indicate that they may play critical roles in vascular biology, such as migration (Table 2).

**TABLE 2:** The effects of TSPANs in vascular cells migration.

TSPANs	Expression	Migration	References
TSPAN2	HUVECs, HCASMCs, MASMCs	HCASMCs↓	Bailey et al., 2011; Zhao et al., 2017
TSPAN27	HUVECs, HDMECs, HRCECs, MLUECs, MLVECs, HCASMCs, MASMCs	HUVECs↓, MLU/VECs↓	Nagao and Oka, 2011; Wei et al., 2014; Zhao et al., 2017
TSPAN8	HUVECs, RAECs, HCASMCs, MASMCs	RAECs↑	Nazarenko et al., 2010; Bailey et al., 2011; Zhao et al., 2017
TSPAN12	HUVECs, HLVECs, MRVECs, HCASMCs, MASMCs	HUVECs↑	Bailey et al., 2011; Bucher et al., 2017; Zhao et al., 2017; Zhang C. et al., 2018
TSPAN24	HUVECs, HLVECs, MLUECs, iBRECs, HDLECs, HMEC-1, HCASMCs, MASMCs	HUVECs↑, MLUECs↑	Yanez-Mo et al., 1998; Deissler et al., 2007; Takeda et al., 2007; Bailey et al., 2011; Iwasaki et al., 2013; Zhao et al., 2017
TSPAN28/30	HUVECs, HLVECs, HSVECs, HMAECs, iBRECs, HDLECs, HCASMCs, MASMCs	HUVECs↑	Yanez-Mo et al., 1998; Klein-Soyer et al., 2000; Deissler et al., 2007; Bailey et al., 2011; Iwasaki et al., 2013; Tugues et al., 2013; Zhao et al., 2017
TSPAN29	HUVECs, HLVECs, HSVECs, HMAECs, iBRECs, HDLECs, HCASMCs, MCASMCs, MASMCs	HUVECs↑, HSVECs↑, HMAECs↑, HCASMCs↑, HDLECs↑, iBRECs↑	Yanez-Mo et al., 1998; Klein-Soyer et al., 2000; Deissler et al., 2007; Kotha et al., 2009; Bailey et al., 2011; Iwasaki et al., 2013; Zhao et al., 2017
TSPAN3/4/5/6/7/9/13/14/18/25/31	HUVECs, HLVECs, HCASMCs, MASMCs	?	Bailey et al., 2011; Zhao et al., 2017
TSPAN11/15/23	HUVECs, HCASMCs, MASMCs	?	Bailey et al., 2011; Zhao et al., 2017
TSPAN17	HLVECs, HCASMCs, MASMCs	?	Bailey et al., 2011; Zhao et al., 2017
TSPAN1/10/20/21/22/26/32/33	HCASMCs, MASMCs	?	Zhao et al., 2017
TSPAN19	HUVECs	?	Bailey et al., 2011

*HUVECs, human umbilical vein endothelial cells; HCASMCs, human coronary artery smooth muscle cells; MASMCs, mouse aortic smooth muscle cells; HDMECs, human dermal microvascular endothelial cells; HRCECs, human retinal capillary endothelial cells; MLUECs, mouse lung endothelial cells; MLVECs, mouse liver endothelial cells; RAECs, rat aortic endothelial cells; HLVECs, human liver endothelial cells; MRVECs, mouse retinal vascular endothelial cells; iBRECs, microvascular endothelial cells of the bovine retina; HDLECs, human dermal lymphatic endothelial cells; HMEC-1, human microvascular endothelial cell line-1; HSVECs, human saphenous vein endothelial cells; HMAECs, human mammary artery endothelial cells; MCASMCs, mouse carotid artery smooth muscle cells.*

TSPAN2 is extensively expressed in medial layer VSMCs of aortas, bladder, brain, lung, skeletal muscle, stomach, heart, spleen, kidney and liver, however, the levels of TSPAN2 is decreased in mouse carotid arteries after ligation injury and in failed human arteriovenous fistula samples after occlusion by dedifferentiated neointimal VSMCs (Zhao et al., 2017). TSPAN2 acts as a suppressor of VSMCs proliferation and migration, and plays important role in the pathogenesis of occlusive vascular diseases (Zhao et al., 2017). Mechanistically, transcription of TSPAN2 in VSMCs is regulated by 2 parallel pathways, TGF-β1/SMAD and myocardin/serum response factor, *via* distinct binding sites in the vicinity of the TSPAN2 promoter (Zhao et al., 2017). Additionally, the single-nucleotide polymorphism located in the regulatory region (G allele at rs12122341) of TSPAN2 is strongly associated with atherosclerosis in large arteries (Ninds Stroke Genetics Network [SIGN] and International Stroke Genetics Consortium [ISGC], 2016). However, the function of TSPAN2 in ECs is not clear.

TSPANC8 subgroup, which have the eight cysteine residues in their large extracellular loops, includes TSPAN5, 10, 14, 15, 17, and 33 (Matthews et al., 2018). TSPAN5 is highly expressed in neocortex, hippocampus, amygdala and in Purkinje cells in the cerebellum of mouse (Garcia-Frigola et al., 2000), and its expression is prominent in both atrial and trabeculated ventricular chambers of the heart on embryonic day 10, indicated that it might be involved in heart development (Garcia-Frigola et al., 2001). TSPAN14 is the major TSPANs of TSPANC8 subgroup in HUVECs and is essential for normal ADAM10 surface expression and activity, while TSPAN33 is the major TSPANs of TSPANC8 subgroup in the erythrocyte lineage and is essential for normal ADAM10 expression (Haining et al., 2012). The vascular functions of TSPANC8 subgroup members still need further investigation.

TSPAN18 is highly expressed in ECs, TSPAN18-knockdown ECs have impaired Ca^2+^ mobilization, and impaired histamine- and thrombin-induced von Willebrand Factor release (Noy et al., 2019). Mechanistically, TSPAN18 interacts with Orai1, which is a major entry route for extracellular Ca^2+^ in non-excitable cells (Noy et al., 2019). Thus, TSPAN18 is essential for Ca^2+^ homeostasis and inflammatory responses in ECs.

Among TSPANs, TSPAN8, TSPAN24, TSPAN12, and TSPAN29 are the main TSPANs family members that facilitate angiogenesis (Hemler, 2014; Bucher et al., 2017; Heo and Lee, 2020). Gesierich et al. (2006) firstly reported that TSPAN8 is the strongest angiogenesis inducer, as that overexpression of TSPAN8 in tumor cells markedly increases angiogenesis *in vivo* and *in vitro*, and co-culture of TSPAN8 knockdown tumor cells or the exosomes-depleted supernatant with HUVECs markedly inhibit HUVECs tube formation *in vitro* (Gesierich et al., 2006; Akiel et al., 2016). Mechanistically, the tumor cells released exosomes containing TSPAN8 are taken up by target cells via ligands for TSPAN8-associated molecules, and induce angiogenic gene transcription and modulate the RNA profile in ECs or adjacent fibroblasts, and exosomes expressing TSPAN8-CD49d complex preferentially bind ECs, thus initiating an angiogenic loop by inducing TSPAN8 itself expression on sprouting ECs (Gesierich et al., 2006; Nazarenko et al., 2010; Mu et al., 2020). The contribution of TSPAN8 and TSPAN24 on angiogenesis has also been confirmed by TSPAN8-KO mice, TSPAN24-KO mice and TSPAN8/24 double-KO mice (Takeda et al., 2007; Zhao et al., 2018a, b) (Supplementary Table 1). Mechanistically, promotion of angiogenesis by tumor-derived exosomes and rescue of impaired angiogenesis in KO mice by wild type-serum exosomes depend on the association of TSPAN8 and TSPAN24 with GPCR and RTK in ECs and tumor cells (Zhao et al., 2018b; Mu et al., 2020). Most importantly, the TSPAN24-integrin complex as a functional unit, and the YRSL motif of TSPAN24 plays key role in TSPAN24-mediated angiogenesis (Sincock et al., 1999; Zhang et al., 2002; Zuo et al., 2010; Liu et al., 2011; Peng et al., 2013; Huang et al., 2016). Based on its effects on angiogenesis, TSPAN24 gene delivery promotes angiogenesis and improves skin temperature in rat hindlimb ischemia model (Liu et al., 2011), enhances myocardial angiogenesis and improves left ventricular function in rat acute myocardial infarction model (Wang et al., 2006; Fu et al., 2015), and the beneficial effect of TSPAN24 on myocardial angiogenesis has also been confirmed in a pig myocardial infarction model (Zuo et al., 2009). In contrast, the oxygen-induced retinal neovascularization and angiogenesis in three tumors models are not decreased in TSPAN24-null mice (Takeda et al., 2007; Deng et al., 2012; Copeland et al., 2013a; Li et al., 2013), indicated that the contributions of TSPAN24 in angiogenesis might be models/diseases dependent. TSPAN24 maintains vascular stability by balancing the forces of cell adhesion and cytoskeletal tension as that TSPAN24 deficiency increases actin cytoskeletal traction by elevating RhoA signaling and diminishes actin cortical meshwork by decreasing Rac1 activity (Zhang et al., 2011). Similar to its influence in angiogenesis, the effect of TSPAN24 on vascular permeability might also be model-dependent as that TSPAN24 deletion did not affect VEGF-induced vascular permeability (Takeda et al., 2011). Moreover, TSPAN24 acts as a molecular linker between MT1-MMP and α3β1 integrin in ECs: MT1-MMP, through its hemopexin domain, associates tightly with TSPAN24, thus forming α3β1 integrin/TSPAN24/MT1-MMP ternary complexes, which is essential for a normal pattern of MT1-MMP-dependent collagenolysis (Yanez-Mo et al., 2008). Thus, TSPAN24 is a key regulator of MT1-MMP in mediating endothelial homeostasis.

TSPAN12, which is expressed in retinal vasculature, has been extensively investigated in ophthalmology (Junge et al., 2009; Nikopoulos et al., 2010; Yang et al., 2011; Seo et al., 2016; Bucher et al., 2017; Lai et al., 2017; Zhang C. et al., 2018). Physiologically, TSPAN12 acts as a key regulator for retinal vascular development by activating NDP- but not Wnt-induced FZD4/β-catenin signaling, early loss of TSPAN12 in ECs causes lack of intraretinal capillaries and increased the expression of ECs-specific adhesion molecule cadherin5, consistent with premature vascular quiescence, late loss of TSPAN12 strongly impairs BRB maintenance without affecting vascular morphogenesis, pericyte coverage, or perfusion, thus, the endothelial TSPAN12 contributes to vascular morphogenesis and BRB formation in developing mice and BRB maintenance in adult mice (Junge et al., 2009; Zhang C. et al., 2018). Mechanistically, TSPAN12 is an essential component of the NDP receptor complex and interacts with FZD4 and NDP *via* its extracellular loops, consistent with an action as co-receptor that enhances FZD4 ligand selectivity for NDP, which signaling is required for normal retinal angiogenesis and BRB function (Lai et al., 2017). Based on its role in retinal angiogenesis during development, mutations in TSPAN12 or large deletions of TSPAN12 cause familial exudative vitreoretinopathy in human (Nikopoulos et al., 2010; Yang et al., 2011; Seo et al., 2016), in contrast, the anti-TSPAN12 antibody, which inhibits ECs migration and tube formation, ameliorates vasoproliferative retinopathy *via* suppressing β-Catenin signaling in rodent models of retinal neovascular disease (Bucher et al., 2017). The function of TSPAN12 in retinal vasculature, especially in retinal ECs, is relatively clear; however, its functions in large arteries are not fully understood.

TSPAN29 promotes angiogenesis and lymphangiogenesis *via* forming functional complexes between VEGFR-3 and integrin α5 and α9, therefore, tumor-induced and inflammation-induced lymphangiogenesis, and tumor-induced angiogenesis are decreased in TSPAN29-KO mice (Iwasaki et al., 2013) (Supplementary Table 1). Intravitreous injections of siRNA-TSPAN29 or anti-TSPAN29 antibodies are therapeutically effective for laser-induced retinal and choroidal neovascularization in mice, and injecting siRNA-TSPAN29 markedly suppresses HGF- or VEGF-induced subconjunctival angiogenesis *in vivo* (Kamisasanuki et al., 2011). Using anti-TSPAN29 monoclonal antibody, three independent experiments revealed that TSPAN29 participates in ECs migration during wound repair *in vitro*, and TSPAN29 is required for platelet-induced HUVECs proliferation (Klein-Soyer et al., 2000; Ko et al., 2006; Deissler et al., 2007). Mechanistically, TSPAN29, TSPAN28, and TSPAN24 localize at cell–cell junctions of ECs and are associated with each other and those of TSPAN29 and TSPAN24 with α3β1 integrin, which are essential for ECs motility, as that monoclonal antibodies directed to both TSPAN24 and TSPAN28 as well as monoclonal antibody specific for α3 integrin, are able to inhibit ECs migration in the process of wound healing (Yanez-Mo et al., 1998). However, another study indicated that ablation of TSPAN29 does not affect proliferation, apoptosis or angiogenesis in primary prostate tumors (Copeland et al., 2013b). Similar to TSPAN24, TSPAN29-dependent angiogenesis might also be model-dependent and perhaps other TSPANs compensates for the absence of TSPAN29 (Hemler, 2014). TSPAN29 also associates with integrins in VSMCs (Scherberich et al., 1998), the neutralization antibody for TSPAN29 reduces the proliferation and migration of VSMCs, and results in a 31% reduction in neointima formation in a mouse carotid ligation injury model, in contrast, overexpression of TSPAN29 leads to 43% increase in neointima (Kotha et al., 2009). To further understand how TSPAN29 regulate adverse VSMCs phenotypes, Herr et al. (2014) used TSPAN29 lentiviral shRNA to knockdown TSPAN29 expression in VSMCs, and found that TSPAN29 deficiency is sufficient to profoundly disrupt cellular actin arrangement and endogenous cell contraction by interfering with RhoA signaling.

In contrast to TSPAN8/12/24/29, ECs TSPAN27 restrains pathologic angiogenesis (Wei et al., 2014). Deficiency of TSPAN27 significantly enhances the migration and invasion of ECs, and markedly increases vascular morphogenesis to various stimuli, however, slightly promotes ECs proliferation and survival (Wei et al., 2014). Mechanistically, TSPAN27 modulates CAMs trafficking by preventing lipid raft aggregation and dissociating CAMs from lipid rafts, and TSPAN27-ganglioside-CD44 signaling restrains angiogenesis by inhibiting ECs adhesiveness and motility (Wei et al., 2014). Therefore, the balance of these TSPANs in regulating angiogenesis is critical for vascular homeostasis.

TSPAN30, which is localized in late endosomes/lysosomes and on the plasma membrane in ECs, contributes to several cell functions relevant to initiation and progression of angiogenesis, such as adhesion and migration of vascular ECs, mechanistically, TSPAN30 associates with both integrin β1 and VEGFR-2 to form functional complexes to modulate VEGFR2 signaling and internalization (Tugues et al., 2013). TSPAN30 also colocalizes with von Willebrand factor and P-selectin to reside in the membrane of Weibel-Palade bodies of ECs (Vischer and Wagner, 1993). Similarly, TSPAN30 coclusters with P-selectin on the plasma membrane of activated ECs, it is thus an essential cofactor to leukocyte recruitment by endothelial P-selectin (Doyle et al., 2011). Other TSPANs are also known to organize leukocyte adhesion molecules into microdomains (Ley, 2011). For example, TSPAN24 and TSPAN29 are components of the endothelial docking structure for adherent leukocytes by their association with ICAM-1 and VCAM-1 in ECs (Barreiro et al., 2005). However, their requirement for leukocyte adhesion is not as stringent as that of TSPAN30 (Ley, 2011). TSPAN28, a putative receptor for hepatitis C virus, is up-regulated in ECs of early atherosclerotic lesions, and it has the potential to substantially enhance monocyte adhesion *via* relocalizing and increasing membrane clustering of ICAM-1 and VCAM-1 (Pileri et al., 1998; Rohlena et al., 2009). Moreover, TSPAN28 interaction with Rac1 through its cytoplasmic C-terminal region limits the GTPase activation within the plasma membrane during cell adhesion and migration (Tejera et al., 2013). As described above, overexpression of TSPAN28 can enhance migrasomes formation in NRK (Huang et al., 2019), however, whether TSPAN28 can influence migrasomes formation in vascular cells has not been investigated.

## Perspective

TSPANs family, which is known to be important in vesicle formation and targeting of vesicles to recipient cell, is involved in a multitude of biological processes, such as development, fertilization, platelet aggregation, parasite and viral infection, immune response induction, metastasis suppression and tumor progression, ophthalmology, synaptic contacts at neuromuscular junctions, maintenance of skin integrity (Hemler, 2003; Levy and Shoham, 2005; Zoller, 2009; Bailey et al., 2011; Rana and Zoller, 2011; Charrin et al., 2014; Colombo et al., 2014; van Niel et al., 2018; Jiang et al., 2019). Moreover, TSPANs are widely expressed in hematopoietic and vascular cells, such as ECs and VSMCs, and are also participated in both physiological and pathological processes related to thrombosis, hemostasis, angiogenesis and vascular injuries (including vascular cells migration), thus emerging novel roles in regulating vascular biology (Zhang et al., 2009; Table 2).

As we have discussed above, migrasomes are organized by TSPANs, cholesterol, integrins and other unidentified molecules, and migracytosis releases cellular contents at a specific location, these cellular contents can be taken up by other cells which travel to that site, indicates that biochemical and spatial information from outgoing cells can be acquired by incoming cells (Ma et al., 2015; Schmidt-Pogoda et al., 2018; Zhao et al., 2019). Considered that migrasomes are distributed in blood vessel, human serum, and in infarcted brain parenchyma of human stroke patients, the contraction and relaxation, vascular repair, and immune responses occurred in blood vessels require vascular cells or immune cells migration, and the localized communication between ECs and VSMCs, and between ECs and immune cells, et al. (Ma et al., 2015; Eelen et al., 2018; Schmidt-Pogoda et al., 2018; Zhao et al., 2019), thus, there is not surprising that migration-dependent migrasomes and migracytosis might play important roles in these processes and act as novel players in mediating vascular homeostasis.

**FIGURE 2:**
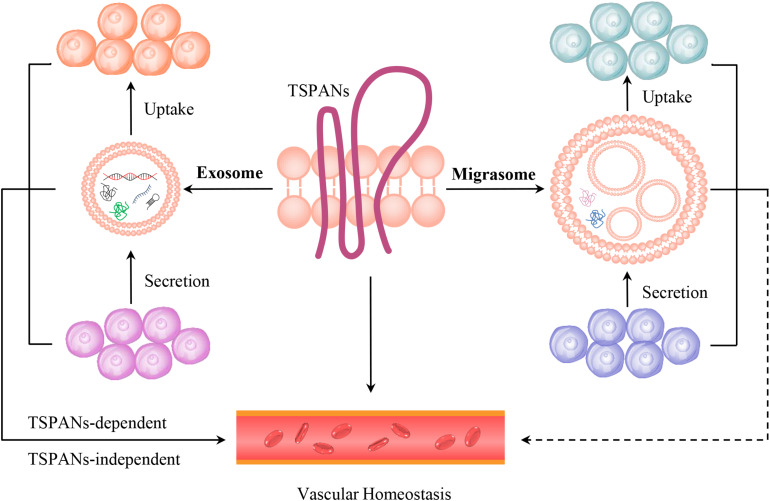
The models of TSPANs, exosomes and migrasomes in vascular homeostasis. TSPANs are the key organizators of both exosome and migrasome. TSPANs can regulate vascualr homeostasis directly. Exosomes, which contain selected proteins, lipids, nucleic acids, amino acids, glycoconjugates and metabolites et al., are secreted by one cell and taken up by another cell, thus transferring signals through cell–cell communication and influencing vascular homeostasis in TSPANs-dependent or independent manners. Migrasomes, which are distributed in blood circulation and blood vessel, contain bioactive cargos, and are also secreted by one cell and taken up by the surrounding cells. Therefore, migrasomes might influence vascular homeostasis *via* cell–cell communication.

It should be noticed that TSPANs are expressed in the membranes of both exosomes and migrasomes (da Rocha-Azevedo and Schmid, 2015; Ma et al., 2015; Ibrahim and Marban, 2016; Wu et al., 2017; Chen et al., 2018, 2019; Schmidt-Pogoda et al., 2018; Huang et al., 2019; Jiang et al., 2019; Pegtel and Gould, 2019; Tavano and Heisenberg, 2019; Zhao et al., 2019). The exosomes play essential roles in ECs dysfunction and regeneration, VSMCs migration, ischemic heart disease, cardiac hypertrophy and fibrosis by transferring their bioactive cargos, such as microRNAs and proteins (Ibrahim and Marban, 2016; Barile et al., 2017; Chang et al., 2018; Tong et al., 2018; Baruah and Wary, 2019; Ranjan et al., 2019). Moreover, cancer cells released exosomes containing TSPAN8 are critical for angiogenesis, as that they can be taken up and initiate angiogenic genes transcription and modulate the RNA profile in ECs or adjacent fibroblasts (Gesierich et al., 2006; Nazarenko et al., 2010; Mu et al., 2020). However, it is not known whether the tumor cells-expressed migraomses can be taken up by ECs *in vivo*, and influence tumor-induced angiogenesis and subsequently tumor growth, and what will happen if these tumor-derived migrasomes reaches organs distant from the tumor? These should be carefully investigated in the near future. Therefore, exosomes- or migrasomes-dependent or independent effects of TSPANs, the TSPANs-dependent or independent effects of exosomes or migrasomes in modulating vascular biology, and the relationships between exosomes and migrasomes in regulating vascular homeostasis should be distinguished and investigated (Figure 2).

The formation of migrasome depends on the matching of specific integrin-ECM pairs, there are 18 α and 8 β integrins in mammals, and each ECM protein has a specific spatial and temporal distribution pattern in a given organism, which will determine when and where migrasome can be generated *in vivo* (Wu et al., 2017). As we have mentioned above, migrasomes display temporal and spatial distributions during the development of zebrafish embryos (Jiang et al., 2019). The percentage of containing in migrasomes is organ-, tissue- or cell type-dependent. Migrasomes in ischemic brain are mainly composed of contractile proteins actin and myosin, cytoskeleton and annexin proteins, while enzymes are the most (31%) contents and much of them are involved in metabolic processes in migrasomes from NRK (Ma et al., 2015; Schmidt-Pogoda et al., 2018; Zhao et al., 2019). The zebrafish embryonic migrasomes are enriched for a host of chemokines, morphogens, cytokines and growth factors, including Tgfβ2, Il1β, PdgfD, Cxcl12b, Wnt11, Mydgf, DllD, Cxcl12a, Bmp1, Wnt8a, Chd, Bmp7a, Cxcl18a.1, Wnt5b, Lefty1 and Bmp2, and migrasomes regulate organ morphogenesis by delivering Cxcl12a/b for Cxcl12a (ligand)-Cxcr4b (receptor) signaling (Jiang et al., 2019). However, the compositions of vascular migrasomes, the origins and targets of migrasomes in human serum, and the pathways of migrasomes entering the circulatory system are not clear (Zhao et al., 2019). Are migrasomes in human serum related to certain cardiovascular diseases, and can they be used as a diagnostic marker (Zhao et al., 2019)? Therefore, these seem that the possible roles of migrasomes and migracytosis in cardiovascular system might be mainly dependent on their origins, compositions, levels, temporal distribution patterns, and locations.

## Author Contributions

YZ and HY contributed to concept and idea. YZ, JZ, and YX prepared the figures and tables. YZ, JW, YD, SZ, and JX wrote the manuscript. All authors have read and agreed to the published version of the manuscript.

## Conflict of Interest

The authors declare that the research was conducted in the absence of any commercial or financial relationships that could be construed as a potential conflict of interest.

## Abbreviations

BRB: blood–retina barrier
CAMs: cell adhesion molecules
circRNA: circular RNA
CPQ: carboxypeptidase Q
dsDNA: double-stranded DNA
ECs: endothelial cells
ECL1: extracellular loop 1
ECM: extracellular matrix
EOGT: EGF domain-specific O-linked *N*-acetylglucosaminetransferase
FZD4: Frizzled-4
GPCR: G-protein-coupled receptor
HCASMCs: human coronary artery smooth muscle cells
HDLECs: human dermal lymphatic endothelial cells
HDMECs: human dermal microvascular endothelial cells
HGF: hepatocyte growth factor
HLVECs: human liver endothelial cells
HMAECs: human mammary artery endothelial cells
HMEC-1: human microvascular endothelial cell line-1
HRCECs: human retinal capillary endothelial cells
HSP: heat shock protein
HSVECs: human saphenous vein endothelial cells
HUVECs: human umbilical vein endothelial cells
iBRECs: microvascular endothelial cells of the bovine retina
ICAM-1: intercellular adhesion molecule-1
KO: knockout
LAMP1: lysosomal associated membrane protein 1
MASMCs: mouse aortic smooth muscle cells
MCASMCs: mouse carotid artery smooth muscle cells
MEF: mouse embryonic fibroblast
miRNA: microRNA
MLUECs: mouse lung endothelial cells
MLVECs: mouse liver endothelial cells
MMP: matrix metalloproteinase
MRVECs: mouse retinal vascular endothelial cells
mtDNA: mitochondrial DNA
mtRNA: mitochondrial RNA
MT1-MMP: membrane-type 1 matrix metalloproteinase
MVB: multivesicular bodies
NDP, norrie disease protein: also referred to as norrin
NDST1: bifunctionalheparan sulfate *N*-deacetylase/*N*-sulfotransferase 1
NeuN: Neuronal Nuclei
NRK: normal rat kidney cell
PDGF-BB: platelet derived growth factor-BB
PIGK, phosphatidylinositol glycan anchor biosynthesis: class K
piRNA: PIWI-interacting RNA
PLSs: pomegranate-like structures
RAECs: rat aortic endothelial cells
RTK: Receptor tyrosine kinase
SAGE: serial analysis of gene expression
SAMs: substrate-attached materials
siRNA-TSPAN29: TSPAN29-specific small interfering RNA
snoRNA: small nucleolar RNA
snRNA: small nuclear RNA
ssDNA: single-stranded DNA
SUMF2: sulfatase modifying factor 2
TEMs: TSPAN-enriched microdomains
TEMAs: tetraspanin- and cholesterol-enriched macrodomains
TM1: transmembrane region 1
tRNA: transfer RNA
TSG101: tumor susceptibility gene 101
TSPANs: tetraspanins
tsRNAs: tRNA-derived small RNAs
VCAM-1: vascular cell adhesion molecule-1
VEGF: vascular endothelial growth factor
VEGFR-3: VEGF receptor 3
VSMCs: vascular smooth muscle cells
WGA: wheat-germ agglutinin.
