# Supplementary material for: Migrasome and Tetraspanins in Vascular Homeostasis: Concept, Present, and Future
Source: Front Cell Dev Biol. 2020 Jun 16;8:438. doi: 10.3389/fcell.2020.00438 (PMC7308473; doi:10.3389/fcell.2020.00438)
Supplement: Supplementary file 1 [file Table_1.DOCX]

Supplementary Table

Table-1. Overview of tetraspanin superfamily

| TSPANs | Aliases in the GenBank | Phenotypes of KO mice |
| --- | --- | --- |
| TSPAN 1 | NET1, TM4C, TM4SF | / |
| TSPAN 2 | NET3, TSN2 | / |
| TSPAN 3 | TM4A, TM4SF8 | TSPAN3^-/-^ mice were born without overt defects, but, TSPAN3 deletion impaired leukemia stem cell self-renewal and disease propagation and markedly improved survival in acute myelogenous leukemia mouse model (Kwon et al., 2015; Seipold et al., 2017). |
| TSPAN 4 | NAG2, TETRASPAN, TM4SF7 | / |
| TSPAN 5 | NET4, TM4SF9 | / |
| TSPAN 6 | T245, TM4SF6 | TSPAN6 deficiency enhanced basal synaptic transmission, impaired long term potentiation (Salas et al., 2017), and altered the amyloid precursor protein processing in neurons (Guix et al., 2017). |
| TSPAN 7 | A15, CCG-B7, CD231, DXS1692E, MRX58, MXS1, TALLA-1, TM4SF2, TM4SF2b | / |
| TSPAN 8 | CO-029, TM4SF3 | Body weight, bone mineral density and phosphorus levels were reduced in male TSPAN8^-/-^ mice (Champy et al., 2011).  The leukocyte migration, wound repair and angiogenesis were defected, and metastasis was impaired in TSPAN8^-/-^, and TSPAN8/CD151 double KO mice (Zhao et al., 2018a). Moreover, the impaired angiogenesis in KO mice can be rescued by wild type-serum exosome (Zhao et al., 2018b). |
| TSPAN 9 | NET5, PP1057 | TSPAN9^-/-^ mice had normal body weights and the normal numbers of blood cells, however, platelets from TSPAN9^-/-^ mice had a mild but specific defect in GPVI-induced platelet aggregation and secretion (Haining et al., 2017). |
| TSPAN 10 | OCSP | / |
| TSPAN 11 | VSSW1971 | / |
| TSPAN 12 | EVR5, NET2, TM4SF12 | TSPAN12^-/-^ mice displayed that the centrifugal outgrowth of the [nerve fiber](https://www.sciencedirect.com/topics/neuroscience/nerve-fiber) layer vasculature was moderately delayed in retinas between postnatal day 5 (P5) and P12 (Junge et al., 2009); at P11, vertical sprouts and outer plexiform layer capillaries were completely absent in TSPAN12*^-/-^* mice, while both were appeared in TSPAN12^+/+^ mice, and the outer plexiform layer remains avascular in adult TSPAN12*^-/-^* mice (Junge et al., 2009); the [thickness](https://www.sciencedirect.com/topics/immunology-and-microbiology/thickness) of the outer nuclear layer in TSPAN12*^-/-^* retinas was consistently reduced in adult but not neonatal mice (Junge et al., 2009). Moreover, formation of microaneurisms, aberrant fenestration, and delayed hyaloid vessel regression are displayed in TSPAN12*^-/-^* mice (Junge et al., 2009). |
| TSPAN 13 | NET6, TM4SF13 | / |
| TSPAN 14 | DC-TM4F2, TM4SF14 | / |
| TSPAN 15 | 2700063A19Rik, NET7, TM4SF15 | / |
| TSPAN 16 | TM-8, TM4-B, TM4SF16 | / |
| TSPAN 17 | FBX23, FBXO23, TM4SF17 | / |
| TSPAN 18 | TSPAN | TSPAN18^-/-^ mice had normal body weights and whole blood cell counts (Noy et al., 2019), and had defective hemostasis due to a defect in non-hematopoietic cells, while deep vein thrombosis and myocardial ischemia/reperfusion injury were improved in these mice (Noy et al., 2019). |
| TSPAN 19 |  | / |
| TSPAN 20 | UPK1B, UPIB, UPK1 | TSPAN20^-/-^ mice exhibited progressive hydronephrosis beginning in adulthood, and possessed unilateral duplex kidneys (Carpenter et al., 2016). |
| TSPAN 21 | UPK1A, UP1A, UPIA, UPKA | / |
| TSPAN 22 | [PRPH2](https://www.ncbi.nlm.nih.gov/gene/5961), AOFMD, AVMD, CACD2, DS, MDBS1, PRPH, RDS, RP7, rd2 | TSPAN22^-/-^ mice failed to form rod outer segments (OSs), and the OSs of TSPAN22^+/-^ mice were short and disorganized (Sanyal et al., 1980; Hawkins et al., 1985; Lee et al., 2006); TSPAN22^-/-^ mice displayed photoreceptor death in animal model of digenic and dominant retinitis pigmentosa (Kedzierski et al., 2001). |
| TSPAN 23 | ROM1, ROM, ROSP1, RP7 | TSPAN23^-/-^ mice formed OSs in which TSPAN22 homotetramers are localized to the disk rims, suggesting that TSPAN22 alone was sufficient for both disk and OS morphogenesis (Clarke et al., 2000). The maximal photoresponse of TSPAN23^-/-^ rod photoreceptors was lower than that of controls (Clarke et al., 2000). |
| TSPAN 24 | CD151, GP27, MER2, PETA-3, RAPH, SFA1 | CD151^-/-^ mice displayed longer average bleeding times, greater average blood loss, and an increased incidence of rebleeding occurrences (Wright et al., 2004); the platelets of CD151^-/-^ mice had impaired outside-in integrin α_IIb_β_3_ signaling (Lau et al., 2004).  CD151^-/-^ mice showed no vascular defects during normal development or during neonatal oxygen-induced retinopathy, while deletion of CD151 resulted in decreased pathologic angiogenesis in other *in vivo* assays and in the *ex vivo* aortic ring assay (Takeda et al., 2007); reduced thrombus growth in FeCl_3_-induced carotid or mesenteric arterioles injury model, laser-induced cremaster muscle arterioles injury model (Orlowski et al., 2009).  Kidney failure in CD151^-/-^ mice, CD151 deficiency resulted in a strain-dependent glomerular disease due to severe alterations of the glomerular basement membrane (Sachs et al., 2006; Baleato et al., 2008; Sachs et al., 2012).  CD151^-/-^ mice were refractory to airway hyperreactivity in response to allergen challenge (Qiao et al., 2017), and these mice spontaneously exhibited age-related pulmonary fibrosis as a result of epithelial disintegrity (Tsujino et al., 2012), however, CD151^-/-^ mice showed markedly diminished lung metastasis (Takeda et al., 2011) and reduced skin tumor formation (Li et al., 2013; Sachs et al., 2014), impaired mammary tumor onset, decreased tumor cell survival, and decreased spontaneous metastasis in ErbB2-transgenic mice (Deng et al., 2012). Similarly, deletion of CD151 reduced mammary tumorigenesis in the mice model of breast cancer induced by the polyoma middle T antigen (PyMT) driven by the murine mammary tumor virus promoter (MMTV) (Roselli et al., 2014).  CD151^-/-^ mice were defective in wound healing (Cowin et al., 2006), and showed exacerbated the IgE-mediated late phase inflammation in a murine model of passive cutaneous anaphylaxis (Abdala-Valencia et al., 2015). |
| TSPAN 25 | CD53, MOX44 | PKC substrate phosphorylation and the recruitment of PKCβ to the plasma membrane were impaired in primary B cells from CD53^-/-^ mice (Zuidscherwoude et al., 2017). |
| TSPAN 26 | CD37, GP52-40 | CD37^-/-^ mice had reduced numbers of IgG-secreting plasma cells in lymphoid organs *via* impairing α(4)β(1) integrin-dependent Akt signaling (van Spriel et al., 2012).  The proliferation of T cells from CD37^-/-^ mice were enhanced, however, antigen-specific T-cell development, T-cell-dependent antibody responses, antitumor immunity were impaired in CD37^-/-^ mice (Knobeloch et al., 2000; van Spriel et al., 2004; Gartlan et al., 2013).  Similar to CD151^-/-^ DCs, DCs from CD37^-/-^ mice were hyper-stimulatory to CD4^+^ and CD8^+^ T cells, although they had different mechanisms (Sheng et al., 2009). However, CD37 deficiency impaired DCs migration, by contrast, DCs from CD82^-/-^ mice displayed a striking hypermigratory phenotype (Gartlan et al., 2013; Jones et al., 2016).  Neutrophil recruitment in a peritonitis model was impaired in CD37^-/-^ mice (Wee et al., 2015).  CD37^-/-^ mice were protected against *Candida albicans* infection, which was accompanied by increased IL-6 levels and IgA antibodies (van Spriel et al., 2009), however, the increased IL-6 levels contributed to glomerular IgA deposition, and the development of renal failure following LPS treatment in CD37^-/-^ mice (Rops et al., 2018). Through constitutive activation of the IL-6 signaling pathway, CD37^-/-^ mice developed germinal center-derived B cell lymphoma in lymph nodes and spleens with a higher incidence than Bcl2 transgenic mice (de Winde et al., 2016). |
| TSPAN 27 | CD82, 4F9, C33, GR15, IA4, KAI1, R2, SAR2, ST6 | CD82^-/-^ mice selectively lose long-term repopulating hematopoietic stem cells (Hur et al., 2016). Similar to CD37^-/-^ mice, T cells from CD82^-/-^ mice were hyperproliferative *in vitro*, however, the Ag-specific CD8^+^ responses in CD82^-/-^ mice were decreased due to the dysfunction of DCs (Jones et al., 2016). CD82^-/-^ mice displayed diminished fungicidal activity, increased *Candida albicans* viability within macrophages, and decreased the production of TNF-α, IL-1β in macrophages (Tam et al., 2019); however, another reports indicated that CD82^-/-^ mice showed an increased survival rate, reduced bacillary loads in the lungs and enhanced inflammation in sera in response to *Mycobacterium tuberculosis* (Koh et al., 2018).  [Global deletion of CD82](https://www.ncbi.nlm.nih.gov/pubmed/29782939) attenuated bone growth, enhanced bone marrow adipogenesis and pathological vascular morphogenesis (Wei et al., 2014; Bergsma et al., 2018). |
| TSPAN 28 | CD81, CVID6, S5.7, TAPA1 | Reduced fertility of female mice lacking CD81 (Rubinstein et al., 2006).  CD81^-/-^ mice fail to develop T helper type 2 immune responses, and diminished allergen-induced airway hyper-reactivity (Maecker et al., 1998; Deng et al., 2000; Deng et al., 2002). *P. yoelii* sporozoites failed to infect CD81-deficient mouse hepatocytes *in vivo* (Silvie et al., 2003).  Increased brain size and glial cell number, but diminished sensitivity to cocaine-induced place preference and increased accumbens dopamine in CD81^-/-^ mice (Michna et al., 2001; Geisert et al., 2002). |
| TSPAN 29 | CD9, BTCC-1, DRAP-27, MIC3, MRP-1 | Deletion of CD9 reduced the number and size of *Propionibacterium acnes*-induced hepatic granulomas (Yamane et al., 2005), diminished inflammation-induced and tumor-induced lymphangiogenesis (Iwasaki et al., 2013), while increased the numbers of multinucleated giant cells in *Propionibacterium acnes*-induced lung inflammation model (Takeda et al., 2003), and enhanced macrophage infiltration and TNFα production in the lung after intranasal administration of LPS (Suzuki et al., 2009).  Similar to that in CD81^-/-^ female mice, female fertility was severely reduced in CD9^-/-^ mice because sperm did not fuse with the oocytes from CD9^-/-^ females (Kaji et al., 2000; Le Naour et al., 2000; Miyado et al., 2000). The fertility of CD9^-/-^ mice is severely but not completely impaired, while CD9 and CD81 double-null mice were completely infertile indicating that CD9 and CD81 play complementary roles in sperm-egg fusion (Rubinstein et al., 2006), moreover, these mice spontaneously developed pulmonary emphysema, developed multinucleated giant cells in the lung, impaired lymphangiogenesis under physiological conditions, and showed enhanced osteoclastogenesis in the bone (Takeda et al., 2003; Takeda et al., 2008; Iwasaki et al., 2013). |
| TSPAN 30 | CD63, LAMP-3, ME491, MLA1, OMA81H | The absence of TSPAN30 resulted in a significant decrease of mast cells degranulation, which translated into a reduction of acute allergic reactions *in vivo* (Kraft et al., 2013). TSPAN30^-/-^ mice showed a significant reduction in both leukocyte rolling and recruitment and a failure of leukocyte extravasation in a peritonitis model (Doyle et al., 2011).  TSPAN30^-/-^ mice displayed an increased urinary flow, water intake and renal Na^+^ excretion; reduced urine osmolality, and higher fecal water content (Schroder et al., 2009; Schulze et al., 2017).  Deficiency of TSPAN30 impaired amyloidogenesis and consequent melanosome maturation *in vivo* (van Niel et al., 2011). TSPAN30^-/-^ mice were less susceptible to hepatic homing and outgrowth of pancreatic ductal adenocarcinoma metastases in the presence of high TIMP1 levels (Grunwald et al., 2016). |
| TSPAN 31 | SAS | / |
| TSPAN 32 | ART1, PHEMX, PHMX, TSSC6 | Similar to CD37^-/-^ mice, TSSC6^-/-^ mice displayed a hyper-proliferative T cell phenotype due to early upregulation of the proliferation-inducing cytokine IL-2 (Tarrant et al., 2002; Gartlan et al., 2010).  TSSC6^-/-^ mice showed features of unstable hemostasis in a tail-bleeding assay, and secondary instability in platelet thrombus formation upon FeCl_3_-induced vascular injury *in vivo* (Goschnick et al., 2006), and clopidogrel-treated TSSC6^-/-^ mice displayed a synergistic defect in unstable haemostasis in this model (Makkawi et al., 2018). Moreover, clopidogrel-treated TSSC6^-/-^ mice showed further synergy with smaller thrombi on collagen under arterial flow (Makkawi et al., 2018). |
| TSPAN 33 | PEN, PEN. | TSPAN33^-/-^ mice developed massive splenomegaly, basophilic macrocytic red blood cells, and anemia as they age (Heikens et al., 2007). |

References

Abdala-Valencia, H., Bryce, P.J., Schleimer, R.P., Wechsler, J.B., Loffredo, L.F., Cook-Mills, J.M., et al. (2015). Tetraspanin CD151 Is a Negative Regulator of FcepsilonRI-Mediated Mast Cell Activation. *J Immunol* 195(4)**,** 1377-1387. doi: 10.4049/jimmunol.1302874.

Baleato, R.M., Guthrie, P.L., Gubler, M.C., Ashman, L.K., and Roselli, S. (2008). Deletion of CD151 results in a strain-dependent glomerular disease due to severe alterations of the glomerular basement membrane. *Am J Pathol* 173(4)**,** 927-937. doi: 10.2353/ajpath.2008.071149.

Bergsma, A., Ganguly, S.S., Dick, D., Williams, B.O., and Miranti, C.K. (2018). Global deletion of tetraspanin CD82 attenuates bone growth and enhances bone marrow adipogenesis. *Bone* 113**,** 105-113. doi: 10.1016/j.bone.2018.05.020.

Carpenter, A.R., Becknell, M.B., Ching, C.B., Cuaresma, E.J., Chen, X., Hains, D.S., et al. (2016). Uroplakin 1b is critical in urinary tract development and urothelial differentiation and homeostasis. *Kidney Int* 89(3)**,** 612-624. doi: 10.1016/j.kint.2015.11.017.

Champy, M.F., Le Voci, L., Selloum, M., Peterson, L.B., Cumiskey, A.M., and Blom, D. (2011). Reduced body weight in male Tspan8-deficient mice. *Int J Obes (Lond)* 35(4)**,** 605-617. doi: 10.1038/ijo.2010.165.

Clarke, G., Goldberg, A.F., Vidgen, D., Collins, L., Ploder, L., Schwarz, L., et al. (2000). Rom-1 is required for rod photoreceptor viability and the regulation of disk morphogenesis. *Nat Genet* 25(1)**,** 67-73. doi: 10.1038/75621.

Cowin, A.J., Adams, D., Geary, S.M., Wright, M.D., Jones, J.C., and Ashman, L.K. (2006). Wound healing is defective in mice lacking tetraspanin CD151. *J Invest Dermatol* 126(3)**,** 680-689. doi: 10.1038/sj.jid.5700142.

de Winde, C.M., Veenbergen, S., Young, K.H., Xu-Monette, Z.Y., Wang, X.X., Xia, Y., et al. (2016). Tetraspanin CD37 protects against the development of B cell lymphoma. *J Clin Invest* 126(2)**,** 653-666. doi: 10.1172/JCI81041.

Deng, J., Dekruyff, R.H., Freeman, G.J., Umetsu, D.T., and Levy, S. (2002). Critical role of CD81 in cognate T-B cell interactions leading to Th2 responses. *Int Immunol* 14(5)**,** 513-523. doi: 10.1093/intimm/14.5.513.

Deng, J., Yeung, V.P., Tsitoura, D., DeKruyff, R.H., Umetsu, D.T., and Levy, S. (2000). Allergen-induced airway hyperreactivity is diminished in CD81-deficient mice. *J Immunol* 165(9)**,** 5054-5061. doi: 10.4049/jimmunol.165.9.5054.

Deng, X., Li, Q., Hoff, J., Novak, M., Yang, H., Jin, H., et al. (2012). Integrin-associated CD151 drives ErbB2-evoked mammary tumor onset and metastasis. *Neoplasia* 14(8)**,** 678-689. doi: 10.1593/neo.12922.

Doyle, E.L., Ridger, V., Ferraro, F., Turmaine, M., Saftig, P., and Cutler, D.F. (2011). CD63 is an essential cofactor to leukocyte recruitment by endothelial P-selectin. *Blood* 118(15)**,** 4265-4273. doi: 10.1182/blood-2010-11-321489.

Gartlan, K.H., Belz, G.T., Tarrant, J.M., Minigo, G., Katsara, M., Sheng, K.C., et al. (2010). A complementary role for the tetraspanins CD37 and Tssc6 in cellular immunity. *J Immunol* 185(6)**,** 3158-3166. doi: 10.4049/jimmunol.0902867.

Gartlan, K.H., Wee, J.L., Demaria, M.C., Nastovska, R., Chang, T.M., Jones, E.L., et al. (2013). Tetraspanin CD37 contributes to the initiation of cellular immunity by promoting dendritic cell migration. *Eur J Immunol* 43(5)**,** 1208-1219. doi: 10.1002/eji.201242730.

Geisert, E.E., Jr., Williams, R.W., Geisert, G.R., Fan, L., Asbury, A.M., Maecker, H.T., et al. (2002). Increased brain size and glial cell number in CD81-null mice. *J Comp Neurol* 453(1)**,** 22-32. doi: 10.1002/cne.10364.

Goschnick, M.W., Lau, L.M., Wee, J.L., Liu, Y.S., Hogarth, P.M., Robb, L.M., et al. (2006). Impaired "outside-in" integrin alphaIIbbeta3 signaling and thrombus stability in TSSC6-deficient mice. *Blood* 108(6)**,** 1911-1918. doi: 10.1182/blood-2006-02-004267.

Grunwald, B., Harant, V., Schaten, S., Fruhschutz, M., Spallek, R., Hochst, B., et al. (2016). Pancreatic Premalignant Lesions Secrete Tissue Inhibitor of Metalloproteinases-1, Which Activates Hepatic Stellate Cells Via CD63 Signaling to Create a Premetastatic Niche in the Liver. *Gastroenterology* 151(5)**,** 1011-1024 e1017. doi: 10.1053/j.gastro.2016.07.043.

Guix, F.X., Sannerud, R., Berditchevski, F., Arranz, A.M., Horre, K., Snellinx, A., et al. (2017). Tetraspanin 6: a pivotal protein of the multiple vesicular body determining exosome release and lysosomal degradation of amyloid precursor protein fragments. *Mol Neurodegener* 12(1)**,** 25. doi: 10.1186/s13024-017-0165-0.

Haining, E.J., Matthews, A.L., Noy, P.J., Romanska, H.M., Harris, H.J., Pike, J., et al. (2017). Tetraspanin Tspan9 regulates platelet collagen receptor GPVI lateral diffusion and activation. *Platelets* 28(7)**,** 629-642. doi: 10.1080/09537104.2016.1254175.

Hawkins, R.K., Jansen, H.G., and Sanyal, S. (1985). Development and degeneration of retina in rds mutant mice: photoreceptor abnormalities in the heterozygotes. *Exp Eye Res* 41(6)**,** 701-720. doi: 10.1016/0014-4835(85)90179-4.

Heikens, M.J., Cao, T.M., Morita, C., Dehart, S.L., and Tsai, S. (2007). Penumbra encodes a novel tetraspanin that is highly expressed in erythroid progenitors and promotes effective erythropoiesis. *Blood* 109(8)**,** 3244-3252. doi: 10.1182/blood-2006-09-046672.

Hur, J., Choi, J.I., Lee, H., Nham, P., Kim, T.W., Chae, C.W., et al. (2016). CD82/KAI1 Maintains the Dormancy of Long-Term Hematopoietic Stem Cells through Interaction with DARC-Expressing Macrophages. *Cell Stem Cell* 18(4)**,** 508-521. doi: 10.1016/j.stem.2016.01.013.

Iwasaki, T., Takeda, Y., Maruyama, K., Yokosaki, Y., Tsujino, K., Tetsumoto, S., et al. (2013). Deletion of tetraspanin CD9 diminishes lymphangiogenesis in vivo and in vitro. *J Biol Chem* 288(4)**,** 2118-2131. doi: 10.1074/jbc.M112.424291.

Jones, E.L., Wee, J.L., Demaria, M.C., Blakeley, J., Ho, P.K., Vega-Ramos, J., et al. (2016). Dendritic Cell Migration and Antigen Presentation Are Coordinated by the Opposing Functions of the Tetraspanins CD82 and CD37. *J Immunol* 196(3)**,** 978-987. doi: 10.4049/jimmunol.1500357.

Junge, H.J., Yang, S., Burton, J.B., Paes, K., Shu, X., French, D.M., et al. (2009). TSPAN12 regulates retinal vascular development by promoting Norrin- but not Wnt-induced FZD4/beta-catenin signaling. *Cell* 139(2)**,** 299-311. doi: 10.1016/j.cell.2009.07.048.

Kaji, K., Oda, S., Shikano, T., Ohnuki, T., Uematsu, Y., Sakagami, J., et al. (2000). The gamete fusion process is defective in eggs of Cd9-deficient mice. *Nat Genet* 24(3)**,** 279-282. doi: 10.1038/73502.

Kedzierski, W., Nusinowitz, S., Birch, D., Clarke, G., McInnes, R.R., Bok, D., et al. (2001). Deficiency of rds/peripherin causes photoreceptor death in mouse models of digenic and dominant retinitis pigmentosa. *Proc Natl Acad Sci U S A* 98(14)**,** 7718-7723. doi: 10.1073/pnas.141124198.

Knobeloch, K.P., Wright, M.D., Ochsenbein, A.F., Liesenfeld, O., Lohler, J., Zinkernagel, R.M., et al. (2000). Targeted inactivation of the tetraspanin CD37 impairs T-cell-dependent B-cell response under suboptimal costimulatory conditions. *Mol Cell Biol* 20(15)**,** 5363-5369. doi: 10.1128/mcb.20.15.5363-5369.2000.

Koh, H.J., Kim, Y.R., Kim, J.S., Yun, J.S., Kim, S., Kim, S.Y., et al. (2018). CD82 hypomethylation is essential for tuberculosis pathogenesis via regulation of RUNX1-Rab5/22. *Exp Mol Med* 50(5)**,** 62. doi: 10.1038/s12276-018-0091-4.

Kraft, S., Jouvin, M.H., Kulkarni, N., Kissing, S., Morgan, E.S., Dvorak, A.M., et al. (2013). The tetraspanin CD63 is required for efficient IgE-mediated mast cell degranulation and anaphylaxis. *J Immunol* 191(6)**,** 2871-2878. doi: 10.4049/jimmunol.1202323.

Kwon, H.Y., Bajaj, J., Ito, T., Blevins, A., Konuma, T., Weeks, J., et al. (2015). Tetraspanin 3 Is Required for the Development and Propagation of Acute Myelogenous Leukemia. *Cell Stem Cell* 17(2)**,** 152-164. doi: 10.1016/j.stem.2015.06.006.

Lau, L.M., Wee, J.L., Wright, M.D., Moseley, G.W., Hogarth, P.M., Ashman, L.K., et al. (2004). The tetraspanin superfamily member CD151 regulates outside-in integrin alphaIIbbeta3 signaling and platelet function. *Blood* 104(8)**,** 2368-2375. doi: 10.1182/blood-2003-12-4430.

Le Naour, F., Rubinstein, E., Jasmin, C., Prenant, M., and Boucheix, C. (2000). Severely reduced female fertility in CD9-deficient mice. *Science* 287(5451)**,** 319-321. doi: 10.1126/science.287.5451.319.

Lee, E.S., Burnside, B., and Flannery, J.G. (2006). Characterization of peripherin/rds and rom-1 transport in rod photoreceptors of transgenic and knockout animals. *Invest Ophthalmol Vis Sci* 47(5)**,** 2150-2160. doi: 10.1167/iovs.05-0919.

Li, Q., Yang, X.H., Xu, F., Sharma, C., Wang, H.X., Knoblich, K., et al. (2013). Tetraspanin CD151 plays a key role in skin squamous cell carcinoma. *Oncogene* 32(14)**,** 1772-1783. doi: 10.1038/onc.2012.205.

Maecker, H.T., Do, M.S., and Levy, S. (1998). CD81 on B cells promotes interleukin 4 secretion and antibody production during T helper type 2 immune responses. *Proc Natl Acad Sci U S A* 95(5)**,** 2458-2462. doi: 10.1073/pnas.95.5.2458.

Makkawi, M., Howells, D., Wright, M.D., and Jackson, D.E. (2018). A complementary role for tetraspanin superfamily member TSSC6 and ADP purinergic P2Y12 receptor in platelets. *Thromb Res* 161**,** 12-21. doi: 10.1016/j.thromres.2017.11.009.

Michna, L., Brenz Verca, M.S., Widmer, D.A., Chen, S., Lee, J., Rogove, J., et al. (2001). Altered sensitivity of CD81-deficient mice to neurobehavioral effects of cocaine. *Brain Res Mol Brain Res* 90(1)**,** 68-74. doi: 10.1016/s0169-328x(01)00092-4.

Miyado, K., Yamada, G., Yamada, S., Hasuwa, H., Nakamura, Y., Ryu, F., et al. (2000). Requirement of CD9 on the egg plasma membrane for fertilization. *Science* 287(5451)**,** 321-324. doi: 10.1126/science.287.5451.321.

Noy, P.J., Gavin, R.L., Colombo, D., Haining, E.J., Reyat, J.S., Payne, H., et al. (2019). Tspan18 is a novel regulator of the Ca(2+) channel Orai1 and von Willebrand factor release in endothelial cells. *Haematologica* 104(9)**,** 1892-1905. doi: 10.3324/haematol.2018.194241.

Orlowski, E., Chand, R., Yip, J., Wong, C., Goschnick, M.W., Wright, M.D., et al. (2009). A platelet tetraspanin superfamily member, CD151, is required for regulation of thrombus growth and stability in vivo. *J Thromb Haemost* 7(12)**,** 2074-2084. doi: 10.1111/j.1538-7836.2009.03612.x.

Qiao, Y., Tam, J.K.C., Tan, S.S.L., Tai, Y.K., Chin, C.Y., Stewart, A.G., et al. (2017). CD151, a laminin receptor showing increased expression in asthmatic patients, contributes to airway hyperresponsiveness through calcium signaling. *J Allergy Clin Immunol* 139(1)**,** 82-92 e85. doi: 10.1016/j.jaci.2016.03.029.

Rops, A., Jansen, E., van der Schaaf, A., Pieterse, E., Rother, N., Hofstra, J., et al. (2018). Interleukin-6 is essential for glomerular immunoglobulin A deposition and the development of renal pathology in Cd37-deficient mice. *Kidney Int* 93(6)**,** 1356-1366. doi: 10.1016/j.kint.2018.01.005.

Roselli, S., Kahl, R.G., Copeland, B.T., Naylor, M.J., Weidenhofer, J., Muller, W.J., et al. (2014). Deletion of Cd151 reduces mammary tumorigenesis in the MMTV/PyMT mouse model. *BMC Cancer* 14**,** 509. doi: 10.1186/1471-2407-14-509.

Rubinstein, E., Ziyyat, A., Prenant, M., Wrobel, E., Wolf, J.P., Levy, S., et al. (2006). Reduced fertility of female mice lacking CD81. *Dev Biol* 290(2)**,** 351-358. doi: 10.1016/j.ydbio.2005.11.031.

Sachs, N., Claessen, N., Aten, J., Kreft, M., Teske, G.J., Koeman, A., et al. (2012). Blood pressure influences end-stage renal disease of Cd151 knockout mice. *J Clin Invest* 122(1)**,** 348-358. doi: 10.1172/JCI58878.

Sachs, N., Kreft, M., van den Bergh Weerman, M.A., Beynon, A.J., Peters, T.A., Weening, J.J., et al. (2006). Kidney failure in mice lacking the tetraspanin CD151. *J Cell Biol* 175(1)**,** 33-39. doi: 10.1083/jcb.200603073.

Sachs, N., Secades, P., van Hulst, L., Song, J.Y., and Sonnenberg, A. (2014). Reduced susceptibility to two-stage skin carcinogenesis in mice with epidermis-specific deletion of CD151. *J Invest Dermatol* 134(1)**,** 221-228. doi: 10.1038/jid.2013.280.

Salas, I.H., Callaerts-Vegh, Z., Arranz, A.M., Guix, F.X., D'Hooge, R., Esteban, J.A., et al. (2017). Tetraspanin 6: A novel regulator of hippocampal synaptic transmission and long term plasticity. *PLoS One* 12(2)**,** e0171968. doi: 10.1371/journal.pone.0171968.

Sanyal, S., De Ruiter, A., and Hawkins, R.K. (1980). Development and degeneration of retina in rds mutant mice: light microscopy. *J Comp Neurol* 194(1)**,** 193-207. doi: 10.1002/cne.901940110.

Schroder, J., Lullmann-Rauch, R., Himmerkus, N., Pleines, I., Nieswandt, B., Orinska, Z., et al. (2009). Deficiency of the tetraspanin CD63 associated with kidney pathology but normal lysosomal function. *Mol Cell Biol* 29(4)**,** 1083-1094. doi: 10.1128/MCB.01163-08.

Schulze, U., Brast, S., Grabner, A., Albiker, C., Snieder, B., Holle, S., et al. (2017). Tetraspanin CD63 controls basolateral sorting of organic cation transporter 2 in renal proximal tubules. *FASEB J* 31(4)**,** 1421-1433. doi: 10.1096/fj.201600901R.

Seipold, L., Damme, M., Prox, J., Rabe, B., Kasparek, P., Sedlacek, R., et al. (2017). Tetraspanin 3: A central endocytic membrane component regulating the expression of ADAM10, presenilin and the amyloid precursor protein. *Biochim Biophys Acta Mol Cell Res* 1864(1)**,** 217-230. doi: 10.1016/j.bbamcr.2016.11.003.

Sheng, K.C., van Spriel, A.B., Gartlan, K.H., Sofi, M., Apostolopoulos, V., Ashman, L., et al. (2009). Tetraspanins CD37 and CD151 differentially regulate Ag presentation and T-cell co-stimulation by DC. *Eur J Immunol* 39(1)**,** 50-55. doi: 10.1002/eji.200838798.

Silvie, O., Rubinstein, E., Franetich, J.F., Prenant, M., Belnoue, E., Renia, L., et al. (2003). Hepatocyte CD81 is required for Plasmodium falciparum and Plasmodium yoelii sporozoite infectivity. *Nat Med* 9(1)**,** 93-96. doi: 10.1038/nm808.

Suzuki, M., Tachibana, I., Takeda, Y., He, P., Minami, S., Iwasaki, T., et al. (2009). Tetraspanin CD9 negatively regulates lipopolysaccharide-induced macrophage activation and lung inflammation. *J Immunol* 182(10)**,** 6485-6493. doi: 10.4049/jimmunol.0802797.

Takeda, Y., He, P., Tachibana, I., Zhou, B., Miyado, K., Kaneko, H., et al. (2008). Double deficiency of tetraspanins CD9 and CD81 alters cell motility and protease production of macrophages and causes chronic obstructive pulmonary disease-like phenotype in mice. *J Biol Chem* 283(38)**,** 26089-26097. doi: 10.1074/jbc.M801902200.

Takeda, Y., Kazarov, A.R., Butterfield, C.E., Hopkins, B.D., Benjamin, L.E., Kaipainen, A., et al. (2007). Deletion of tetraspanin Cd151 results in decreased pathologic angiogenesis in vivo and in vitro. *Blood* 109(4)**,** 1524-1532. doi: 10.1182/blood-2006-08-041970.

Takeda, Y., Li, Q., Kazarov, A.R., Epardaud, M., Elpek, K., Turley, S.J., et al. (2011). Diminished metastasis in tetraspanin CD151-knockout mice. *Blood* 118(2)**,** 464-472. doi: 10.1182/blood-2010-08-302240.

Takeda, Y., Tachibana, I., Miyado, K., Kobayashi, M., Miyazaki, T., Funakoshi, T., et al. (2003). Tetraspanins CD9 and CD81 function to prevent the fusion of mononuclear phagocytes. *J Cell Biol* 161(5)**,** 945-956. doi: 10.1083/jcb.200212031.

Tam, J.M., Reedy, J.L., Lukason, D.P., Kuna, S.G., Acharya, M., Khan, N.S., et al. (2019). Tetraspanin CD82 Organizes Dectin-1 into Signaling Domains to Mediate Cellular Responses to Candida albicans. *J Immunol* 202(11)**,** 3256-3266. doi: 10.4049/jimmunol.1801384.

Tarrant, J.M., Groom, J., Metcalf, D., Li, R., Borobokas, B., Wright, M.D., et al. (2002). The absence of Tssc6, a member of the tetraspanin superfamily, does not affect lymphoid development but enhances in vitro T-cell proliferative responses. *Mol Cell Biol* 22(14)**,** 5006-5018. doi: 10.1128/mcb.22.14.5006-5018.2002.

Tsujino, K., Takeda, Y., Arai, T., Shintani, Y., Inagaki, R., Saiga, H., et al. (2012). Tetraspanin CD151 protects against pulmonary fibrosis by maintaining epithelial integrity. *Am J Respir Crit Care Med* 186(2)**,** 170-180. doi: 10.1164/rccm.201201-0117OC.

van Niel, G., Charrin, S., Simoes, S., Romao, M., Rochin, L., Saftig, P., et al. (2011). The tetraspanin CD63 regulates ESCRT-independent and -dependent endosomal sorting during melanogenesis. *Dev Cell* 21(4)**,** 708-721. doi: 10.1016/j.devcel.2011.08.019.

van Spriel, A.B., de Keijzer, S., van der Schaaf, A., Gartlan, K.H., Sofi, M., Light, A., et al. (2012). The tetraspanin CD37 orchestrates the alpha(4)beta(1) integrin-Akt signaling axis and supports long-lived plasma cell survival. *Sci Signal* 5(250)**,** ra82. doi: 10.1126/scisignal.2003113.

van Spriel, A.B., Puls, K.L., Sofi, M., Pouniotis, D., Hochrein, H., Orinska, Z., et al. (2004). A regulatory role for CD37 in T cell proliferation. *J Immunol* 172(5)**,** 2953-2961. doi: 10.4049/jimmunol.172.5.2953.

van Spriel, A.B., Sofi, M., Gartlan, K.H., van der Schaaf, A., Verschueren, I., Torensma, R., et al. (2009). The tetraspanin protein CD37 regulates IgA responses and anti-fungal immunity. *PLoS Pathog* 5(3)**,** e1000338. doi: 10.1371/journal.ppat.1000338.

Wee, J.L., Schulze, K.E., Jones, E.L., Yeung, L., Cheng, Q., Pereira, C.F., et al. (2015). Tetraspanin CD37 Regulates beta2 Integrin-Mediated Adhesion and Migration in Neutrophils. *J Immunol* 195(12)**,** 5770-5779. doi: 10.4049/jimmunol.1402414.

Wei, Q., Zhang, F., Richardson, M.M., Roy, N.H., Rodgers, W., Liu, Y., et al. (2014). CD82 restrains pathological angiogenesis by altering lipid raft clustering and CD44 trafficking in endothelial cells. *Circulation* 130(17)**,** 1493-1504. doi: 10.1161/circulationaha.114.011096.

Wright, M.D., Geary, S.M., Fitter, S., Moseley, G.W., Lau, L.M., Sheng, K.C., et al. (2004). Characterization of mice lacking the tetraspanin superfamily member CD151. *Mol Cell Biol* 24(13)**,** 5978-5988. doi: 10.1128/MCB.24.13.5978-5988.2004.

Yamane, H., Tachibana, I., Takeda, Y., Saito, Y., Tamura, Y., He, P., et al. (2005). Propionibacterium acnes-induced hepatic granuloma formation is impaired in mice lacking tetraspanin CD9. *J Pathol* 206(4)**,** 486-492. doi: 10.1002/path.1793.

Zhao, K., Erb, U., Hackert, T., Zoller, M., and Yue, S. (2018a). Distorted leukocyte migration, angiogenesis, wound repair and metastasis in Tspan8 and Tspan8/CD151 double knockout mice indicate complementary activities of Tspan8 and CD51. *Biochim Biophys Acta Mol Cell Res* 1865(2)**,** 379-391. doi: 10.1016/j.bbamcr.2017.11.007.

Zhao, K., Wang, Z., Hackert, T., Pitzer, C., and Zoller, M. (2018b). Tspan8 and Tspan8/CD151 knockout mice unravel the contribution of tumor and host exosomes to tumor progression. *J Exp Clin Cancer Res* 37(1)**,** 312. doi: 10.1186/s13046-018-0961-6.

Zuidscherwoude, M., Dunlock, V.E., van den Bogaart, G., van Deventer, S.J., van der Schaaf, A., van Oostrum, J., et al. (2017). Tetraspanin microdomains control localized protein kinase C signaling in B cells. *Sci Signal* 10(478). doi: 10.1126/scisignal.aag2755.
